# Skin metastasis of lung carcinoma

**DOI:** 10.11604/pamj.2015.21.13.6843

**Published:** 2015-05-07

**Authors:** Krich Sanaa, Mernissi Fatima Zahra

**Affiliations:** 1 Dermatological Department, Hassan II University Hospital, Fes, Morocco

**Keywords:** Metastasis, lung, cancer

## Image in medicine

A 61-year-old male was admitted to the hospital with tumors in the scalp (A), face (B), and neck (C) that had been present for four month with an acute decrease in visual acuity. These tumors have progressed to ulceration. Clinical examination observed although limited 3 lumps varying sizes from 1,5mm to 2 cm and ulcerated at the center. Dermoscopy was objective polymorphic vessels. The differential diagnosis of keratoacanthoma, epidermoid carcinoma, melanoma, primary adnexal tumour and skin infections was considered. Biopsies of the tumor in the scalp revealed moderately-differentiated epidermoid carcinoma, which were considered to be cutaneous metastases. Cerebral magnetic resonance imaging and thoracoabdominal pelvic scanner objectivated a tumoral mass of lung (D) with ocular, hepatica and bone metastasis. Lung biopsy was objectived a Squamous cell carcinoma. The diagnosis of Skin metastasis of lung carcinoma was made and and he is currently under undergoing Chemotherapy. Cutaneous metastasis is caused by primary cancer-derived cells that grow in the skin. The diagnosis of tumour metastatic to the skin is not usually difficult, but identification of the primary source of the metastasis can sometimes be a problem. According to the published literature, the incidence of cutaneous metastasis is 2.9-5.3% in general, and 1-12% for lung cancer. Certain cases may also manifest as erysipelas-like cancer, vascular dilation or bullous-like lesions, papules, plaques or scarring.

**Figure 1:**
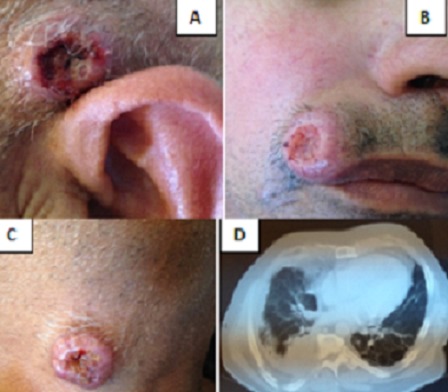
(A) ulcerated tumors in the scalp; (B) face; (C) neck; (D) tumoral mass of lung

